# Local blockage of self-sustainable erythropoietin signaling suppresses tumor progression in non-small cell lung cancer

**DOI:** 10.18632/oncotarget.19354

**Published:** 2017-07-18

**Authors:** Lei He, Shouzhen Wu, Qiang Hao, Elhadji M. Dioum, Kuo Zhang, Cun Zhang, Weina Li, Wei Zhang, Yingqi Zhang, Jiming Zhou, Zhijun Pang, Lijuan Zhao, Xiaowen Ma, Meng Li, Qiuyang Zhang

**Affiliations:** ^1^ State Key Laboratory of Cancer Biology, Biotechnology Center, School of Pharmacy, The Fourth Military Medical University, Xi’an, China; ^2^ Shaanxi Institute of Pediatric Diseases, Xi’an Children’s Hospital, Xi’an, China; ^3^ Department of Internal Medicine, The University of Texas Southwestern Medical Center, Dallas, Texas, USA; ^4^ Current/Present address: Diabetes Department, Nestle Institute of Health Science, EPFL Campus, Lausanne, Switzerland; ^5^ Current/Present address: Center for Esophageal Research, Baylor University Medical Center, Dallas, Texas, USA

**Keywords:** serum EPO, cell cycle, proliferation, NSCLC, hypoxia

## Abstract

Functional significance of co-expressed erythropoietin (EPO) and its receptor (EPOR) in non-small cell lung cancer (NSCLC) had been under debate. In this study, co-overexpression of EPO/EPOR was confirmed to be positively associated with poor survival in NSCLC. The serum EPO in 14 of 35 enrolled NSCLC patients were found elevated significantly and decreased to normal level after tumor resection. With primary tumor cell culture and patient-derived tumor xenograft (PDX) mouse model, the EPO secretion from the tumors of these 14 patients was verified. Then, we proved the patient derived serum EPO was functionally active and had growth promotion effect in EPO/EPOR overexpressed but not in EPO/EPOR under-expressed NSCLC cells. We also illustrated EPO promoted NSCLC cell proliferation through an EPOR/Jak2/Stat5a/cyclinD1 pathway. In xenograft mouse model, we proved local application of EPO neutralizing antibody and short hairpin RNA (shRNA) against EPOR effectively inhibited the growth of EPO/EPOR overexpressed NSCLC cells and prolonged survivals of the mice. Finally, EPO/EPOR/Jak2/Stat5a/cyclinD1 signaling was found to be a mediator of hypoxia induced growth in EPO/EPOR overexpressed NSCLC. Our results illustrated a subgroup of NSCLC adapt to hypoxia through self-sustainable EPO/EPOR signaling and suggest local blockage of EPO/EPOR as potential therapeutic method in this distinct NSCLC population.

## INTRODUCTION

Erythropoietin (EPO), a glycoprotein produced in the fetal liver and the adult kidney, is the chief regulator of erythropoiesis [1]. In adult organs, EPO is also involved in injury repair and tissue regeneration through EPO receptor (EPOR) located in target organs [2–4]. Over the last decade, growing evidences have shown the co-expression of EPO and EPOR are connected to cancer cell growth, migration, and invasiveness in various human malignancies [5–9]. Moreover, a number of studies have demonstrated recombinant human EPO (rhEPO), also known as erythropoiesis stimulating agents (ESAs) which have been widely used to relieve chemotherapy-induced anemia may enhance tumor progression or decrease patient survival [10–13]. Nonetheless, the negative impact of EPO in cancer patients is controversial or not straightforward [14, 15]. For instance, in a preclinical myeloma model, rhEPO induced tumor regression and antitumor immune responses [16]. In a separate study however, EPO does not affect breast tumor cells but promote self-renewal of tumor-initiating cell [17].

Non-small cell lung cancer (NSCLC) is a leading cause of worldwide cancer death [18]. Although platinum-based combination chemotherapy and additional treatment methods have made modest progress in NSCLC, the overall prognosis of NSCLC patients is still discouraging [19, 20]. A better understanding of biological mechanisms involved in NSCLC development is needed. Like other cancer types, co-expression of EPO and EPOR has been reported in NSCLC and was associated with poor survival of NSCLC patients [21–25]. However, several studies have denied EPOR expression and functionality in NSCLC [26–28]. So far, this disagreement remains unchanged. In addition, the underlying molecular mechanism of EPO mediated effects has never been reported in NSCLC.

In this study, we confirmed the correlation between EPO/EPOR overexpression and poor prognosis in NSCLC using paraffin-embedded specimens and tissue microarrays (TMA). We then designed top-down experiments to assess the EPO secretion, function, and underlying mechanism using human blood and tumor specimens, primary tumor cell culture, patient-derived tumor xenograft (PDX) mouse model, NSCLC cell lines and xenograft mouse model. Furthermore, we explored the possibility of using EPO signaling as therapeutic targets in NSCLC. Finally, we characterized the role of EPO/EPOR in hypoxia induced tumor progression.

## RESULTS

### EPO/EPOR expression was associated with tumor progression and overall survival in NSCLC

Sixty formalin-fixed and paraffin-embedded (FFPE) specimens of human NSCLC were used to assess EPO and EPOR expression by immunohistochemistry (IHC). EPOR staining was positive in 93.3% (56 of 60) of tumors and among them, 41.6% (25 of 60) were strongly positive (2+). EPO staining was positive in 60.3% (38 of 60) and among them, 35% (21 of 60) were strongly positive (2+) (Supplementary Figure 1A). All of the 21 strong EPO positive specimens (2+) were also found to be strong EPOR-positive (2+). To determine the clinical relevance of tumor EPO and EPOR expression, we investigated the association of EPO and EPOR expression with several clinical parameters. No difference in EPOR expression was observed according to age, gender, smoking history, histological classification and tumor stage. On the other hand, EPO expression was significantly higher in stage III and IV patients than stage II patients (*P*=0.035), and in patients with smoking history compared to nonsmokers as well (*P*=0.001) (Supplementary Table 1).

Next, we determined if EPO and EPOR expression were associated with the overall survival based on TMA IHC staining. Strongly positive expression (2+) was found in 38% (57 of 150) of TMA tissue spots for EPO and 42.6% (64 of 150) for EPOR. Concurrent strong expression (2+) of EPO and EPOR accounted for 36% (54 of 150) of TMA tissue spots which was consistent with the results from FFPE specimens. The single strong positive group had a poorer five-year overall survival, compared to their corresponding negative/moderate expression group (*P*=0.11 for EPOR and *P*=0.06 for EPO, Supplementary Figure 1B and 1C). The double strong positive group had a significant poorer five-year overall survival (*P*=0.021, Supplementary Figure 1D).

### Serum EPO was elevated in a subgroup of NSCLC patients with a direct link to tumor load

**Figure 1:**
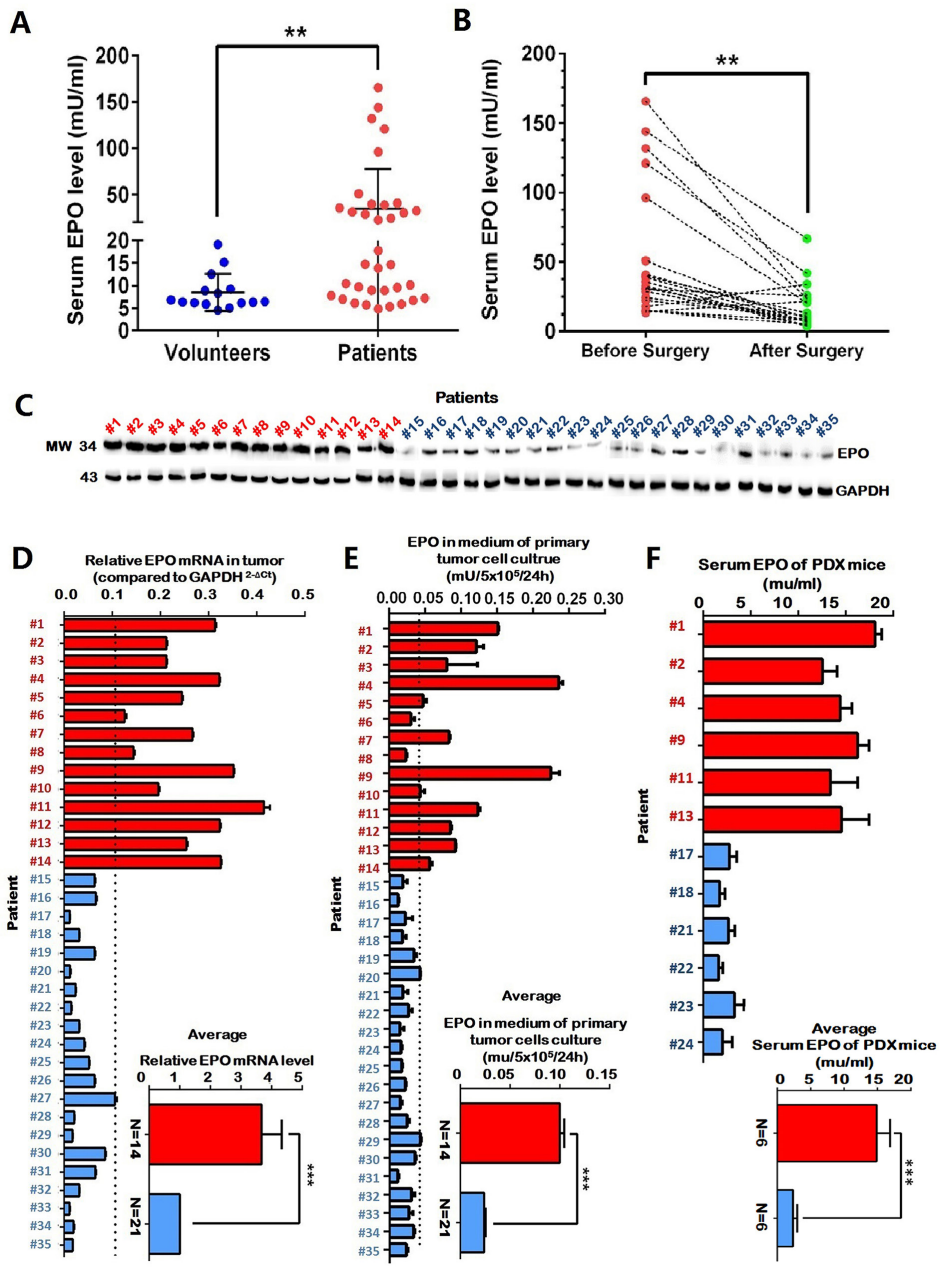
Elevated serum erythropoietin (EPO) was associated with tumor load in a subgroup of NSCLC patients **(A)** The average EPO level of NSCLC patients (N=35) was significantly higher than that of healthy volunteers (N=15). **(B)** In a subgroup of NSCLC patients (n=16) whose EPO levels higher than any of the healthy volunteers, 14 patients had serum EPO dropped significantly after tumor resection (paired t-test). **(C** and **D)** Intracellular EPO mRNA and protein expression in the tumors of the 14 patients whose serum EPO dropped after tumor resection (red), was significantly higher than those of other patients (blue). **(E)** Significantly higher EPO concentrations were present in the supernatant of the primary tumor cell cultures of the 14 patients whose serum EPO dropped after tumor resection (red) compared with those of other patients (blue). **(F)** In the patient-derived tumor xenograft (PDX) model, mice with high-EPO tumors (N=6, red) had significant higher serum EPO concentration compared to the mice with low EPO tumors (N=6, blue). The inserted panels in D, E and F showed mean values of the two subgroup patients. Serum and medium EPO were determined by enzyme-linked immunosorbent assay (ELISA). EPO expressions were determined by quantitative RT-PCR and Western blot. Mean±SD, ***, p≤0.001; **, p≤0.01.

Serum EPO levels in 35 stage-II NSCLC patients and 15 healthy volunteers were examined by ELISA. Before surgeries, the average EPO level of NSCLC patients was significantly higher than that of healthy volunteers (*P*=0.002). Sixteen of the 35 patients had serum EPO levels above the highest value of healthy volunteers (Figure 1A). Three weeks after tumor resection, serum EPO of these 16 patients were measured again. Among them, the serum EPO of 14 patients dropped significantly after surgeries (*P*=0.005). The serum EPO in the remaining two patients increased slightly (Figure 1B). The average EPO level of pre-surgery samples (61.15±18.79) was significantly higher than that of post-surgery samples (15.87±3.74) (*P*=0.002). Significant differences were found in various strata regarding clinical parameters including age, gender, smoking history and histology (Table 1).

**Table 1:** Demographic and clinical characteristics of 16 NSCLC patients with abnormal serum EPO concentrations

	No. of patients (n)	Serum EPO (mU/ml) before surgery	Serum Epo (mU/ml) after surgery	*P* value
**All patients**	16	61.15±18.79	13.87±3.74	0.018
**Age**				
62<	6	30.8±4.86	6.79±1.45	0.000
62≥	10	81.39±30.22	18.59±5.37	0.007
**Gender**				
male	9	78.36±32.45	16.23±6.17	0.041
female	7	40.11±12.41	11.00±3.73	0.019
**Smoking status**				
non-smoker	4	49.11±19.36	12.42±6.08	0.043
current or ex-smoker	12	65.19±26.00	14.51±4.8	0.035
**Histology**				
adenocarcinoma	8	46.15±13.79	11.99±3.52	0.016
squamous cell carcinoma	8	74.59±35.63	15.78±6.78	0.043

Comparison made with the paired t-test. All p values are two-sided. Cut off value of age is median value.

To exclude the possibility of EPO elevation in patients was due to impaired pulmonary functions, tobacco use or other life styles, we examined the EPO expression in excised tumors using western blot and qRT-PCR. The tumors from the 14 patients whose serum EPO dropped significantly after tumor resections had higher EPO expression compared to the tumors from the other 21 patients (Figure 1C and 1D). Furthermore, the supernatants of the primary tumor cell cultures from these 14 patients had higher EPO concentrations than those from the other 21 patients (Figure 1E). PDX mice with high-EPO tumors (N=6) had significantly higher serum EPO concentration compared to the mice with low-EPO tumors (N=6) (Figure 1F). Using serum from healthy mice, we confirmed that the human EPO ELISA kit in our experiments did not detect mouse EPO (data not shown).

### Serum EPO from the patients was functionally active in EPO/EPOR overexpressed NSCLC cells

We surveyed EPO and EPOR expression in nine NSCLC cell lines with normal bronchial epithelial cells HBEC-3KT and EPO-dependent leukemia cells UT-7 included as controls. Compared to HBEC-3KT and UT-7, the NSCLS cells H1155, H1819, H1833 and H3122 expressed significantly higher levels EPO and EPOR than other NSCLC cells (Supplementary Figure 2A). Two EPO/EPOR high expressing cells (H1155, H1819) and one EPO/EPOR low expressing (HCC15) were used for subsequent experiments.

**Figure 2:**
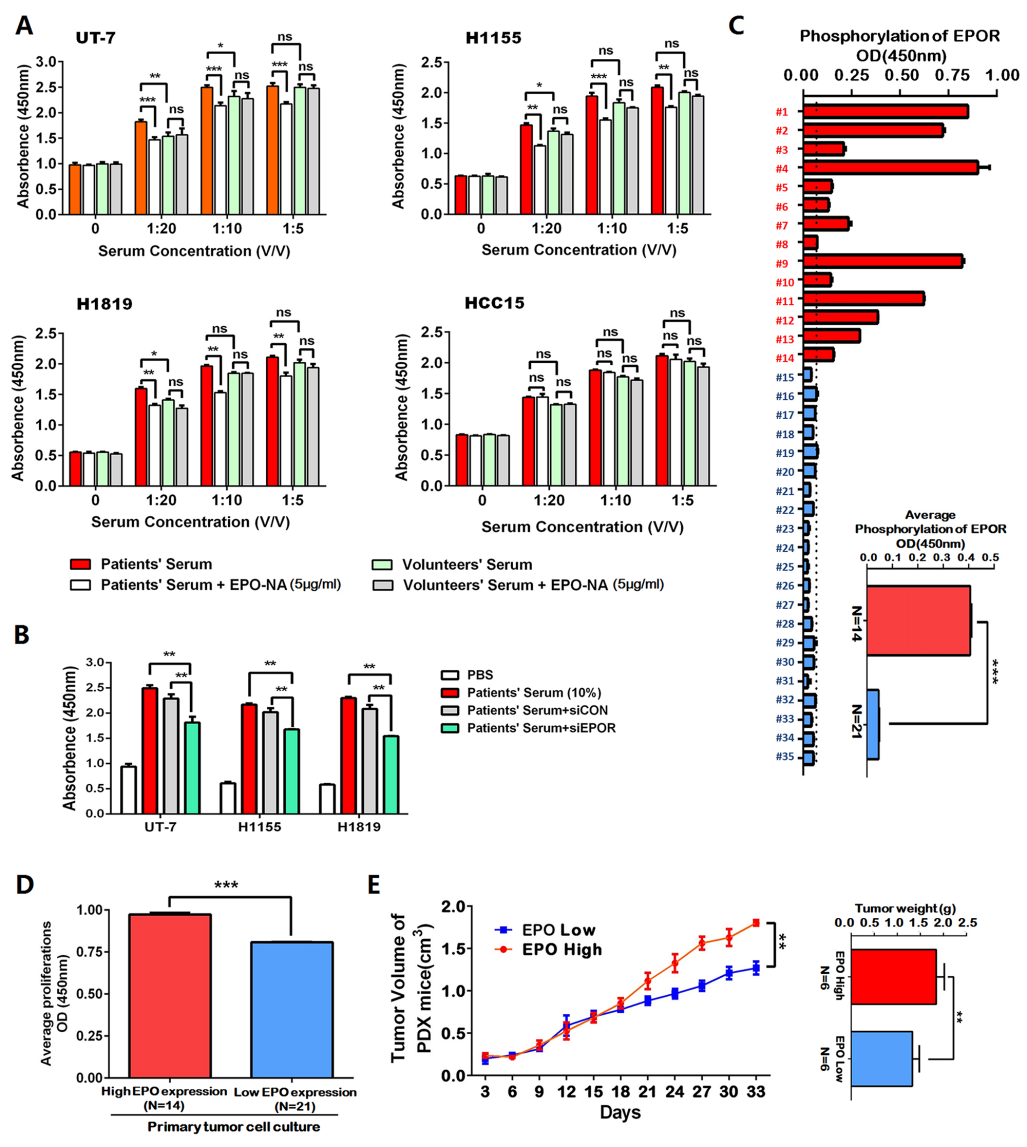
The erythropoietin (EPO) in the patients’ sera was functionally active in EPO/EPOR overexpressed NSCLC cells **(A)** Treatments using the sera from the above 14 patients who had higher EPO level (before surgery) as well as sera from health volunteers resulted in a significant increase of cell proliferation in the H1155, H1819 and HCC15 cells in a dose-dependent manner. The error bars represent the SD of 14 different experiments. UT-7 cells were included as a positive control. In EPO/EPOR overexpressed cells (UT-7, H1155 and H1819), the growth-promoting effects of the patients’ serum were blocked partially by EPO neutralizing antibody (EPO-NA, 5μg/ml), while no such blocking effects were observed for the volunteers’ sera. In HCC15 cells which are EPO/EPOR low-expressed, EPO-NA did not interrupt the growth promotion effects induced by the patients’ sera. **(B)** Compared with control siRNA (siCON), the siRNA against EPOR (siEPOR) significantly inhibited the growth promoting effects induced by patients’ sera in H1155, H1819 and UT-7 cells. **(C)** Enzyme-linked immunosorbent assay (ELISA) showed the phosphorylated EPOR (p-EPOR) in the above higher EPO expressing tumor specimens (N=14, red) was significantly higher than those in other tumor specimens (N=21, blue). The inserted panel showed the mean values of two patient subgroups. **(D)** The average cell proliferation index of primary cultured cells from the above tumors with higher EPO expression (red) was higher than that with lower EPO expression (blue). **(E)** The patient derived tumor xenograft (PDX) mice with high EPO tumors (N=6, red) exhibited increased growths of xenografts compared with the mice with low EPO tumors (N=6, blue). Mean±SD; ***, p≤0.001; **, p≤0.01; ns, not significant.

Treatments using the sera from the above 14 patients who had higher EPO level (before surgery) as well as sera from health volunteers resulted in a significant increase in cell proliferation of UT-7, H1155, H1819 and HCC15 in a dose-dependent manner. At a lower concentration (1:20), the patients’ sera had greater growth-promoting effect on EPO/EPOR overexpressed cells (H1155, H1819 and UT-7) than volunteers’ sera. EPO neutralizing antibody (EPO-NA, 5μg/ml) partially blocked the growth-promoting effects of the patients’ sera but not that of the volunteers’ sera in H1155, H1819 and UT-7 cells. In HCC15 cells, the promotions of the patients’ sera and volunteers’ sera had no significant difference and EPO-NA did not show significant interruptions (Figure 2A). To determine this promotion is mediated by EPOR, we transfected UT-7, H1819 and H1155 cells with siRNA against EPOR (siEPOR) or control siRNA (siCON) (Supplementary Figure 2B). Compared with siCON, siEPOR significantly inhibited the growth promoting effects induced by patients’ sera (Figure 2B). To further prove the function of serum EPO in patients, the expression levels of the phosphorylated EPOR (pEPOR) in the tumors from above 35 patients were investigated using ELISA. The results showed the pEPOR levels in EPO-elevated tumors (N=14) were significantly higher than those in other tumor specimens (N=21) (Figure 2C). Moreover, the average cell proliferation index of primary cell cultures from the tumors with higher EPO expression was higher than that from other tumors (Figure 2D). The high-EPO PDX tumors (N=6) grew faster in mice compared with the low-EPO PDX tumors (N=6, Figure 2E).

Next, we repeated all treatments using recombinant human (rh) EPO. The rhEPO resembled the growth-promoting effects of patients’ sera on UT-7, H1155 and H1819 cells, which was totally blocked by EPO-NA or siEPOR. The rhEPO had no significant growth-promoting effects in HCC15 (Supplementary Figure 3A). In our experiments, rhEPO did not appear to protect against etoposide-induced apoptosis (Supplementary Figure 3B) or promote cell migration in all cell lines (Supplementary Figure 3C).

### EPO/EPOR promoted cell cycle through Jak2/Stat5a/cyclinD1 signaling in NSCLC

To explore the mechanism underlying the growth-promoting effects of EPO/EPOR in NSCLC cells, we examined the changes in cell cycles. The rhEPO treatment enhanced the cell cycle of H1819, H1155 and UT-7 as shown by significant decreased percentage of G1-phase cells and increased percentage of S-phase cells. This cell cycle-promoting effect of rhEPO was diminished in the presence of EPO-NA (Supplementary Figure 4A) or siRNA against EPOR (Supplementary Figure 4B). Treatment by the NSCLC patient serum promoted cell cycle in the way similar to those by rhEPO, which was partially abolished by siEPOR (Supplementary Figure 4C).

**Figure 3:**
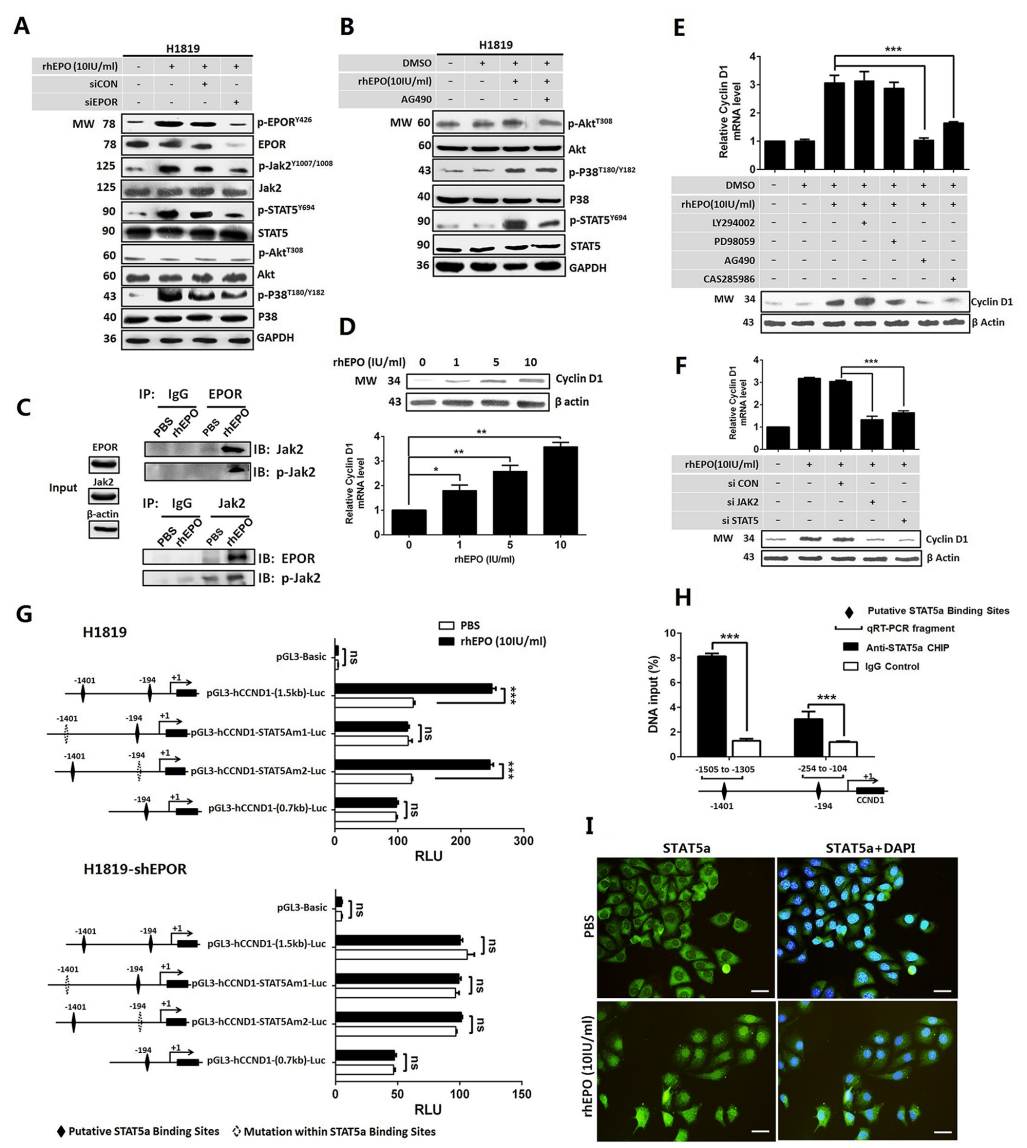
EPO/EPOR promoted cell cycle through Jak2/Stat5a/cyclinD1 signaling in NSCLC **(A)** Recombinant human (rh) EPO treatment induced the phosphorylation of EPOR, JAK2, and downstream molecules STAT5 and P38 but not AKT. These effects were suppressed by siRNA against EPOR (siEPOR). **(B)** AG490, a JAK2 inhibitor, blocked rhEPO-induced STAT5 activation but did not affect p38 and AKT activation. **(C)** Immunoprecipitation with the antibodies of JAK2 or EPOR followed by western blotting for phosphorylated-JAK2 (p-JAK2) confirmed rhEPO induced the interaction of EPOR/JAK2 and the following JAK2 phosphorylation. **(D)** Real-time PCR (lower panel) and western blot (upper panel) showed rhEPO treatment increased cyclin D1 mRNA and protein expression. **(E)** Treatment by JAK2 (AG490) or STAT5 inhibitor (CAS285986) but not PI3K/AKT (LY294002) or p38 MAPK inhibitor (PD98059) abrogated rhEPO-induced cyclin D1 mRNA (upper panel) and protein (lower panel) expression. **(F)** Treatment by siRNAs against JAK2 or STAT5 diminished rhEPO induced cyclin D1 mRNA (upper panel) and protein (lower panel) expression. **(G)** Reporter assay showed rhEPO significantly increased the luciferase activity of the constructs containing wild-type CCND1 promoter in parental H1819 cells (upper panel). The putative STAT5a-binding element located at the upstream of transcription initiation site (-1401) was essential to this rhEPO-mediated promoter activity. The rhEPO did not induce luciferase activity of any of constructs in EPOR shRNA stably transfected H1819 cells (lower panel). **(H)** Chromatin immunoprecipitation assay indicated that a STAT5a antibody was able to pull down the DNA fragments containing two putative STAT5a binding elements (-1401 and -194). The enrichment of the -1401-containing fragments was higher than that of the -194-containing fragments. **(I)** Confocal microscopy showed rhEPO treatment induced the nuclear translocation of STAT5a. GAPDH or β-actin was included as loading control in western blot, and β-actin was used as a reference gene in real-time PCR. Bar=20μm; All *in vitro* experiments were performed in triplicate. Mean±SD; ***, p≤0.001; **, p≤0.01; *, p≤0.05; ns, not significant.

In the process of erythropoiesis, EPO/EPOR signals through JAK2/STAT pathway involving STAT5, STAT3 or STAT2. We therefore asked which STAT molecule plays essential role in cell cycle regulation mediated by EPO in NSCLC cells. In H1819, only the phosphorylation level of STAT5 increased after rhEPO treatment (Supplementary Figure 5A). In addition to using STAT as the direct transcription factor, activation of JAK2/STAT could also trigger PI3K/AKT or RAS/RAF/ERK downstream pathway. In H1819 cells, we found rhEPO induced the phosphorylation of EPOR, JAK2, and downstream molecules STAT5 and p38 but not AKT. These effects were suppressed by siRNA against EPOR (Figure 3A). AG490 (a JAK2 inhibitor) treatment resulted in the blockage of rhEPO-induced STAT5 activation but did not affect p38 and AKT activation (Figure 3B). These results suggested JAK2/STAT5 was the main downstream factors of EPO/EPOR in NSCLC cells. Furthermore, immunoprecipitation with the antibodies of JAK2 or EPOR followed by western blotting for phosphorylated-JAK2 (p-JAK2) confirmed that rhEPO induced the interaction of EPOR/JAK2 and consequentially increased JAK2 phosphorylation (Figure 3C).

Since we had proved EPO/EPOR signaling promoted G1 to S phase transition in cell cycle, we next determined whether activation of EPOR/JAK2/STAT5 was associated with cyclin D or cyclin A expression. By qRT-PCR and Western Blot we showed rhEPO treatment increased cyclin D1 (Figure 3D) but not other D-cyclin and cyclin A (Supplementary Figure 5B). The rhEPO-induced cyclin D1 expression was abrogated by JAK2 inhibitor (AG490) or STAT5 inhibitor (CAS285986) but not by PI3K/AKT inhibitor (LY294002) or p38 MAPK inhibitor (PD98059) (Figure 3E). Similar results were found from the cells treating by JAK2 or STAT5 siRNA (Figure 3F).

To determine if the increased cyclin D1 mRNA expression was regulated at transcriptional level, we identified two putative STAT5a-binding elements within the 1.5-kb CCND1 promoter fragment using online algorithms (-1401 and -194, Supplementary Figure 5C). No STAT5b-binding elements were identified within this promoter region. To determine if STAT5a mediated rhEPO-induced cyclin D1 promoter activity, H1819 cells were transfected with wild-type as well as mutated cyclin D1 promoter-luciferase constructs followed by treating cells with or without rhEPO. The rhEPO induced a 2-fold increase in luciferase activity with a full-length wild-type CCND1 promoter, while this rhEPO-induced promoter activity was abolished with site-directed mutagenesis at -1401 but not at -194 of the putative STAT5a-binding motifs. A construct contained truncated cyclin D1 promoter (absence of the -1401 site) had also lost the response to rhEPO treatment (Figure 3G upper panel). In addition, in the H1819 cells stably transfected with EPOR shRNA (Supplementary Figure 6A), rhEPO treatment did not induce luciferase activity of any of these constructs (Figure 3G lower panel). Chromatin immunoprecipitation (ChIP) assay showed that a STAT5a antibody was able to pull down the DNA segments containing putative binding elements. The amount of the -1401 segment pulled down by the antibody was higher than that of the -194 segment (Figure 3H). Next, we showed nuclear translocation of STAT5a after rhEPO treatment by confocal microscopy, which further demonstrated that the activation of JAK2/STAT5a was functionally relevant (Figure 3I). Western blotting with antibodies specifically against phosphorylated STAT5a (p-STAT5a^Y694^) and STAT5b (p-STAT5b^S731^) demonstrated rhEPO did not induce the activation of STAT5b (Supplementary Figure 6B).

### Inhibition of EPO/EPOR signaling suppressed the proliferation of EPO addicted NSCLS cells *in vivo*

**Figure 4:**
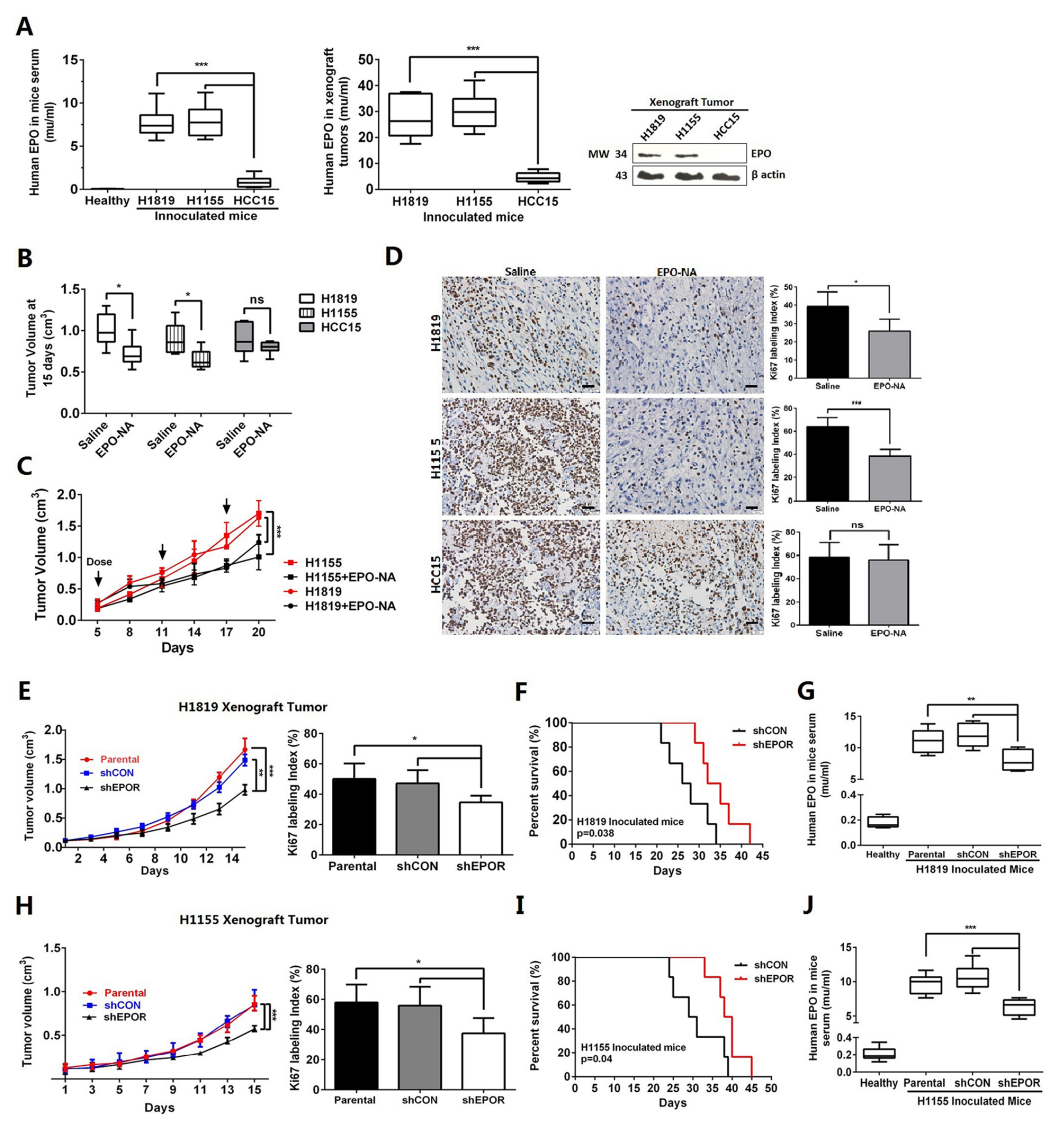
EPO/EPOR inhibition suppressed proliferation of EPO addicted NSCLS cells *in vivo* **(A)** Significantly higher EPO levels were present in the sera and tumor masses of the H1819 and H1115 xenograft mice than those of HCC15. **(B** to **D)** Administration of EPO neutralizing antibody (EPO-NA) to xenograft tumor areas significantly reduced the final tumor volumes **(B)**, delayed tumor growth **(C)**, and decreased Ki67 labeling **(D)** in the H1819 and H1155 xenograft tumors. **(E** to **J)** In mice inoculated with stable EPOR-knockdown H1819 (H1819-shEPOR) or H1155 cells (H1155-shEPOR), the growth of the xenograft tumors were significantly delayed with reduced Ki67 labeling index at harvest **(E and H)**. These mice also exhibited longer survival time **(F and I)**. The H1819-shEPOR **(G)** and H1155-shEPOR **(J)** xenograft mice had significantly reduced human EPO in serum compared with the mice inoculated with corresponding parental tumor cells. Six mice were assigned to each treatment group. Bar=20μm; Mean±SD; ***, p≤0.001; **, p≤0.01; *, p≤0.05; ns, not significant.

H1155, H1819 or HCC15 cells were inoculated into nude mice subcutaneously. When all tumors grew up to about 1-cm^3^ (N=10), human EPO levels in mouse serum and xenograft tumor mass were determined separately by ELISA and western blot. Compared with HCC15 xenograft mice, the H1819 or H1115 xenograft mice had significantly higher levels of human EPO in serum and in tumor masses (Figure 4A). Administration of EPO-NA to xenograft tumor areas significantly decreased the H1819 and H1155 tumor burdens after 15 days (Figure 4B) and caused a delayed growth of established tumors (Figure 4C). EPO-NA treated H1819 and H1155 xenograft tumors also had reduced Ki67 labeling indexes compared with untreated tumors (Figure 4D). Moreover, the phosphorylation of STAT5a and the expression of cyclin D1 in EPO-NA treated H1819 and H1155 tumors were significantly reduced compared with normal saline treated ones (Supplementary Figure 6C). For HCC15, EPO-NA treatment neither inhibited tumor growth nor reduced Ki67 labeling (Figure 4B and 4D).

Furthermore, we established stably transfected clones with shRNA against EPOR in H1819 and H1155 cells (shEPOR) (Supplementary Figure 6A). In nude mice, H1819-shEPOR cells showed a significant delay of tumor growth and declined Ki67 labeling index compared with parental cells or control shRNA transfected cells (shCON) (Figure 4E). Moreover, the mice with H1819-shEPOR tumors exhibited a prolonged survival time (*P*=0.038, Figure 4F). In addition, mice with H1819-shEPOR xenograft tumors had significantly reduced human EPO in serum (Figure 4G) suggesting the delay of tumor growth led to the decrease of endogenous EPO. Similar results were found in mice with H1155-shEPOR xenograft tumors (Figure 4H-4J). These results suggest the possibility of therapeutically targeting EPO signaling in NSCLC, and suggested blocking access to EPOR in tumor area may be helpful when rhEPO were used for chemotherapy-related anemia.

### EPO/EPOR/Jak2/Stat5a/cyclinD1 signaling was a mediator of hypoxia induced cell growth in EPO/EPOR overexpressed NSCLC

**Figure 5:**
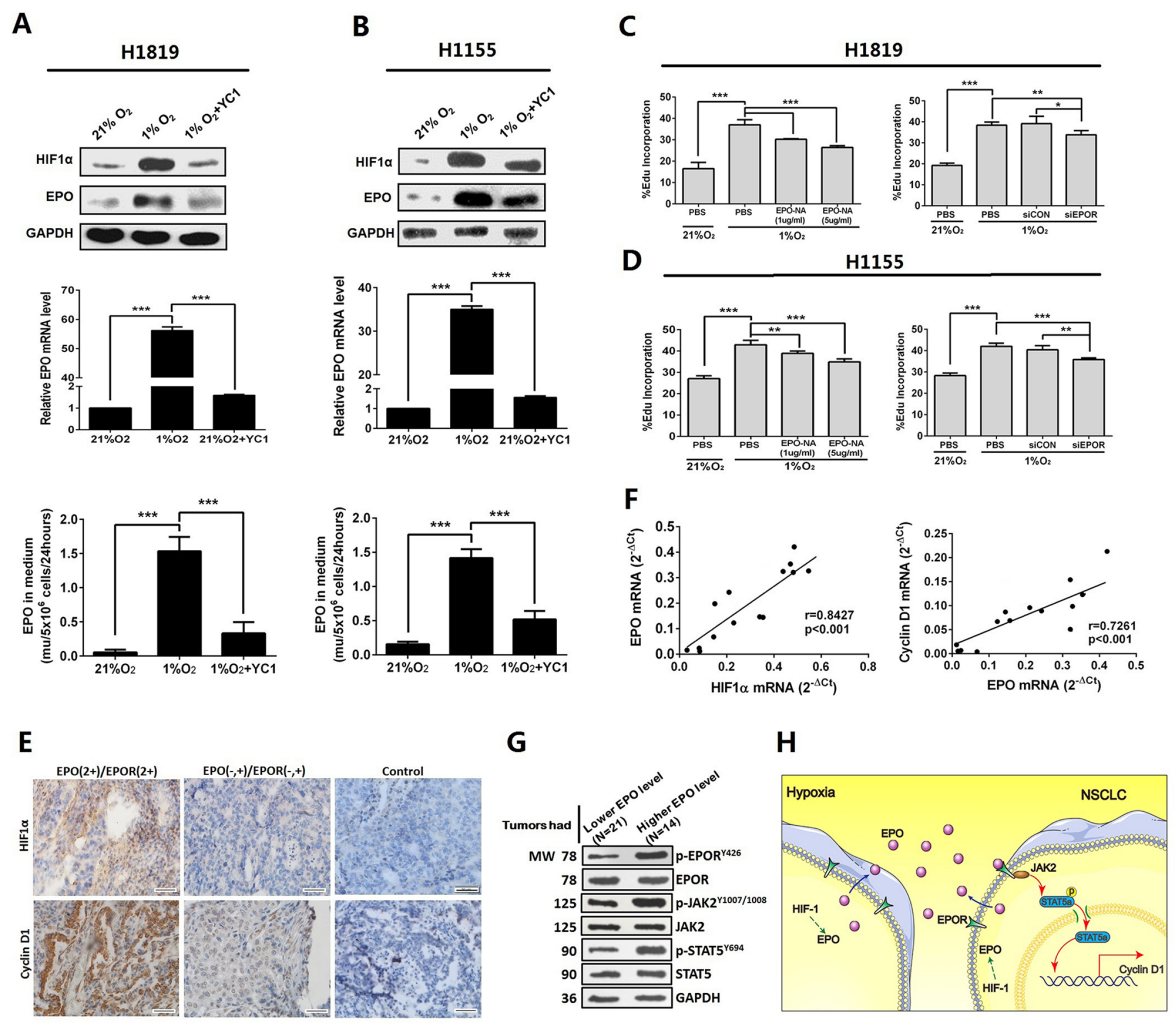
EPO/EPOR/Jak2/Stat5a/cyclinD1 signaling was a mediator of hypoxia induced cell growth in NSCLC **(A** and **B)** Hypoxia significantly increased the expression and secretion of EPO in H1819 and H1155 cells. Pretreatment by YC-1, a hypoxia inducible factor 1α (HIF-1α) inhibitor, diminished these effects. **(C** and **D)** Treatment by EPO neutralizing antibody (EPO-NA) or EPOR siRNA (siEPOR) reduced hypoxia-induced proliferation in H1819 and H1155 cells. **(E)** Representative images showed immunohistochemistry staining of HIF-1α and cyclin D1 in the 60 FFPE specimens of NSCLC. N=39 for the EPO negative (-) and moderate (+) group; N=21 for the EPO 2+/EPOR 2+ group; control was normal IgG staining. **(F)** Significant positive correlation was found between HIF-1α and EPO mRNA expression (left panel) and between EPO and cyclin D1 mRNA expression (right panel) in the tumor tissues from the 14 patients whose serum EPO dropped after tumor resection. **(G)** Expression of phosphor (p)-EPOR, p-JAK2 and p-STAT5 were higher in tumor tissues from the patients with high serum EPO (N=14) than those from the patients (N=21) whose serum EPO was at low level or remained unchanged after surgery. **(H)** A schematic model summarizing our findings: NSCLC cells overexpressed and secreted EPO under hypoxic stress which promotes cell proliferation via NSCLC cell surface EPOR and downstream JAK2/STAT5/Cyclin D1 signaling. Bar=20μm; Mean±SD; ***, p≤0.001; **, p≤0.01; *, p≤0.05; ns, not significant.

Hypoxia is an important initiator of tumorigenesis and progression in solid tumors, and EPO is a well-established hypoxia-inducible gene. We assessed expression and secretion of EPO in NSCLC cells under 21% and 1% O_2_ exposure. To determine the contribution of autocrine/paracrine EPO to hypoxia-induced proliferation, the NSCLC cells were pre-treated with EPO-NA or siEPOR before hypoxic exposure. We found the expressions and secretions of EPO were hypoxia-inducible in H1819 and H1155 cells, which was diminished by YC1, a hypoxia inducible factor 1α (HIF-1α) inhibitor (Figure 5A and 5B). In addition, both EPO-NA and EPOR siRNA reduced hypoxia-induced proliferation in H1155 and H1819 cells (Figure 5C and 5D). These results suggest hypoxic stress in tumors can induce autocrine EPO/EPOR signaling to promote cell proliferation in NSCLC.

To verify our findings from the cultured cells, we examined expressions of HIF-1α, JAK2, STAT5 and Cyclin D1 in the initial sixty FFPE NSCLC specimens. Compared with EPO negative (-) and moderate (+) specimens (n=39), the expressions of HIF-1α and cyclin D1 in EPO (2+)/EPOR (2+) specimens (n=21) were at higher levels (Figure 5E). Next, we examined mRNA expression of HIF1α, EPO and Cyclin D1 in the tumor tissues from the initial 14 patients whose serum EPO dropped after tumor resections. Significant positive correlation was found between HIF-1α mRNA and EPO mRNA expression (Two sided Pearson’s correlation, r=0.843, p<0.001), and between EPO and Cyclin D1 mRNA expression (r=0.726, p<0.001) (Figure 5F). Furthermore, the phosphorylation levels of EPOR and STAT5 in these 14 tumor specimens were significantly increased compared to that in the remaining 21 tumor specimens from the patients whose serum EPO was at low level or remained unchanged after surgery (Figures 1 and 5G). A schematic model summarizing our findings is provided in Figure 5H.

## DISCUSSION

Although EPO and EPOR have been found expressed in NSCLC, their value as potential prognostic marker in NSCLC are still under debate [21, 22, 24–27]. For instance, Saintigny et al. showed that co-expression of EPO and EPOR is associated with poor survival in stage I NSCLC [24]. In contrast, Rózsás et al. have found high EPOR level as a potential positive prognostic marker in human lung adenocarcinoma [21]. To unravel this difference and to get a definitive understanding for this scenario especially in Chinese population, we assessed EPO and EPOR expression and clinical relevance using 60 FFPE NSCLC specimens and a TMA containing 150 spotted NSCLC samples. We found that the dual strong EPO and EPOR positive was associated with shorter overall survival, which is consistent with those reported by Saintigny et al. [24]. We also found that the EPO expression is positively correlated with disease stages. Our findings are better controlled because our results were built upon the entire EPO signaling axis and our clinical analysis was based on EPO as well as EPOR data. In comparison, the works by Rózsás et al. was only based on the analysis of EPOR expression which may not be adequate [21].

In human malignancy, growing evidences suggested that EPO is produced and secreted by cancer cells [17, 29–31]. In this study, the analysis of serum EPO from NSCLC patients and from mouse models supported that EPO was indeed secreted from high EPO-expressing NSCLC tumor cells. We also illustrated hypoxia was an inducer of EPO secretion from NSCLC cells. Moreover, in the dual EPO and EPOR positive tumor cells the tumor-derived EPO was functionally active that was evidenced by: (1) the blockage of EPO or knockdown of EPOR diminished the growth promotion effects of the NSCLC patients’ sera *in vitro*, and (2) the xenograft tumors of stable EPOR knocked-down cells or with local administration of EPO-NA exhibited delayed growth *in vivo*. To our best knowledge, this is the first demonstration of autocrine/paracrine EPO signaling in NSCLC both *in vitro* and *in vivo*.

It is reported that activated EPOR signals through JAK2 and downstream STAT5, PI3K/AKT or RAS/RAF/ERK to promote pro-proliferative or anti-apoptotic cancer cell growth [7, 32]. In this study, we identified JAK2/STAT5a as the sole downstream pathway of EPOR in NSCLC. Meanwhile, cyclin D1 was identified as the key molecule of EPO function for the first time. We determined that the EPO/EPOR signaling promoted cell cycle but not cell migration or anti-apoptosis. In comparison, Merkle et al. found that rhEPO can inhibit cisplatin-induced apoptosis in lung cancer cells [27]. We attribute this inconsistency to different sensibility to EPO and chemotherapy drugs between their and our cells. In contrast to Merkle’s and our studies, Rózsás et al. reported that rhEPO did not alter the growth of lung adenocarcinoma cells *in vitro* and decreased tumor growth *in vivo* [21]. We noticed that Rózsás et al. used a dose of 1 to 3 IU/ml rhEPO which is much lower than those used in previous reports as well as in this study [17, 33]. In addition, Rózsás et al. did not examine the EPO expression levels in their cell lines. This may have caused the major discrepancy between their and our studies because as we showed in this study, EPO expression may determine whether the EPO/EPOR signaling network is active in these cells.

Rózsás et al. showed that the inhibitory effect of rhEPO in xenograft tumor was due to the stimulation of angiogenesis which in turn brings more chemotherapeutic drugs to tumor masses. However, systematic administration of rhEPO in xenograft mice to address tumorigenic effect of endogenous EPO is inappropriate because that may confound its pro-tumor effects by other affected organs and systems such as hematopoietic and immune systems. In this study, we did not observe changes in tumor capillary densities after local EPO blockage or EPOR knockdown (data not shown). Angiogenesis provides nutrient support to cancer cells and enables self-sufficient tumor growth and therefore, has become a well-known therapeutic target [34, 35]. The rhEPO is also reported to promote lung cancer growth by stimulating angiogenesis [36]. Thus, whether the rhEPO-induced tumor angiogenesis is an advantage or disadvantage still needs more investigation.

Although it is generally disputable on whether ESAs treatment is a benefit or harm to the progression-free and overall survival of NSCLC patients [28, 37], the results of our study confirmed the role of endogenous EPO in lung tumorigenesis and cautioned the adverse impacts of ESAs at least in a subgroup of NSCLC patients. Our data suggested that under previous clinical trials, the patients should have been evaluated for EPO and EPOR expression before enrollment, and the effect of ESAs should be evaluated between the subgroups of low and high EPO/EPOR-expressing patients. Finally, our results suggest blocking the access to EPOR on tumor cells during ESAs treatment may be helpful to prevent tumorigenicity and not to affect erythropoiesis.

In summary, we have illustrated EPO could be directly secreted from the tumors of a subgroup of NSCLC patients, and the tumor derived EPO was capable of promoting the dual EPO and EPOR-positive NSCLC progression. Local blockage of EPO signaling could suppress the growth of dual EPO and EPOR-positive NSCLC tumor and prolong survivals of xenograft mice. EPO promoted NSCLC cell proliferation solely depending on an EPOR/Jak2/Stat5a/cyclin D1 pathway. Self-sustainable EPO/EPOR signaling was a mediator of hypoxia induced cell growth in dual EPO and EPOR-positive NSCLC tumor. In general, our study illustrated a subgroup of NSCLC can adapt to tumor microenvironment through EPO signaling. Clinically, our data support a rationale for local blockage of EPO/EPOR signaling as potential therapeutic method in EPO/EPOR overexpressed NSCLC.

## MATERIALS AND METHODS

### Clinical samples

35 NSCLC patients and 15 healthy volunteers were enrolled to evaluate serum EPO level in the Department of Thoracic Surgery (Tangdu Hospital, The Fourth Military Medical University, Xi’an, China). All 35 patients were histologically confirmed to have stage II NSCLC according to the WHO criteria and the tumor-node-metastasis classification. None of the patients received neoadjuvant chemotherapy and ESAs before surgery. All patients were free of the bone marrow or kidney diseases that can induce abnormal EPO level. In addition, 60 FFPE specimens of pathologically confirmed NSCLC and related clinical information were obtained from the archived tissue bank in the Department of Pathology (Xijing Hospital, The Fourth Military Medical University). A TMA containing 150 NSCLC samples and corresponding adjacent non-cancerous normal lung tissues were purchased from OUTDO BIOTECH (Shanghai, China). Five year survival Information of the related patients was also provided by OUTDO BIOTECH. The study was approved by the ethics committee of the Fourth Military Medical University and all patients signed written informed consents that were subjected to approval of the institutional review board before study procedures.

### Cell culture

Normal human bronchial epithelial cells (HBEC-3KT) and NSCLC cell lines (HCC15, H44, H2073, H1993, H1155, H1819, H1833, H3122) were provided by Dr. John Minna (University of Texas Southwestern Medical Center, Dallas, TX). NSCLC cell line A549 was purchased from the American Type Culture Collection (Manassas, VA). The human EPO-dependent leukemia cell line (UT-7) was purchased from China Center for Type Culture Collection (Shanghai, China). All cell lines were authenticated by short tandem repeat profiling (Microread Genetics, Shanghai, China) and passaged for less than 6 months. HBEC-3KT cells were cultured in Keratinocyte-serum free medium with a supplementation of Epidermal Growth Factor, Bovine Pituitary Extract and gentamycin (GIBCO/Invitrogen, Carlsbad, CA). A549 were cultured in DMEM/F12 (1:1) medium (GIBCO) supplemented with 10% fetal bovine serum (FBS) and antibiotics. Other cells were cultured in RPMI 1640 medium (GIBCO) with 10% FBS and antibiotics.

For primary cell culture, fresh tumors were minced and digested with Type IV Collagenase (Life Technologies, Gaithersburg, MD) for 3 hours min at 37°C. Single-cell suspensions were obtained by filtration through a strainer (70 μm, BD Biosciences, Bedford, MA). Dead cells and red blood cells were removed using the Ficoll gradient centrifugation method and ACK (Ammonium-Chloride-Potassium) lysis buffer (Thermo Fisher Scientific, Waltham, MA). Then, primary tumor cells were cultured in DMEM/F12 medium (GIBCO) supplemented with 10% FBS. All cells were maintained at 37°C with a supply of 5% CO_2_.

### Enzyme-linked immunosorbent assay (ELISA)

EPO levels were assayed in triplicate using a human ELISA kit (R&D, Minneapolis, MN) following the manufacturer’s instructions. Sample preparations depended on what material was used. Serum was separated from blood samples using a pipette after the blood clotted (4°C, overnight) and stored at-80 °C until use. For secreted EPO from primary tumor cells or NSCLC cell lines, 5×10^6^ cells were plated in 10 cm dishes with 5 ml of serum-free medium and cultured in 1% O_2_ incubator for 24 hours. Media were then collected and concentrated to 200 μl using centrifugal filter units (Merck Millipore, Billerica, MA). For EPOR phosphorylation detection, two different species derived antibodies against EPOR antibody or phosphorylated EPOR (Abcam, Cambridge, UK) were used.

### Mouse models

Male athymic nude mice (6-8 weeks) were purchased from Experimental Animal Center of The Fourth Military Medical University. For PDX mouse model, necrotic and supporting tissues were removed from the fresh tumor tissues using a scalpel. Twenty microgram tissue fragments were implanted subcutaneously into the flank region of nude mice using a trocar. All tissues were kept on wet ice and engrafted within 12 hours after resection. Suture and antibiotics were used if necessary. For xenograft model of cell lines, 1×10^5^ cells were re-suspended in 100 μl of HBSS/Matrigel (1:1) (BD Biosciences), mixed, and subcutaneously (s.c.) injected into the lower flank of the mice. All tumors were monitored and measured with digital calipers every day. For EPO neutralization, saline or EPO neutralizing antibody (EPO-NA, 2μg/mice/dose, AB-286-NA, R&D) was injected into tumor locations every 5 days. No further treatment was applied to the mice inoculated with EPOR knocked-out cells. All animal work was performed in accordance with protocols approved by the Animal Care and Use Committee of the university. Mice were maintained in accordance with the guidelines of the Chinese Public Health Service Policy on Human Care and Use of Laboratory Animals.

### Luciferase reporter assay

We identified potential binding sites for transcription factor STAT5 in the promoter region of Cyclin D1 gene (CCND1) based on three computer algorithms (Jaspar: http://jaspar.genereg.net/, PROMO:http://alggen.lsi.upc.es/cgi-bin/promo_v3/promo/promoinit.cgi?dirDB=TF_8.3 and TFSEARCH: http://www.cbrc.jp/research/db/TFSEARCH.html). A 1.5 kb fragment of the CCND1 promoter was amplified and subcloned into KpnI and XhoI sites of the pGL3 basic vector (Promega, Madison, WI) to yield pGL3-hCCND1-(1.5 kb)-Luc construct. Using pGL3-hCCND1p-(1.5 kb)-Luc as template, a 5’-truncated fragments (-664 to +37nt) were obtained by PCR, and two site-directed mutations within the STAT5a-binding elements at -1401 and -194 of CCND1 promoter were introduced with QuikChange Site-directed Mutagenesis Kit (Stratagene, La Jolla, CA). The resulting fragments were cloned into pGL3 vector to yield pGL3-hCCND-(0.7kb)-Luc, pGL3-hCCNDm1-Luc and pGL3-hCCNDm2-Luc constructs. H1819 cells were transfected with pGL3 basic vector or above CCND1 constructs using Lipofectamine 2000 Reagent (Life Technologies). After growth arrest in serum-free medium for 24h, the cells were treated with or without rhEPO (10IU/ml) for 12 h. After washing with cold PBS, lysed in NP40 buffer and centrifugation, the cell extracts were assayed for luciferase activity using the Luciferase Assay System (Promega) and a single tube luminometer (TD20/20 Turner Designs, Sunnyvale, CA). The values are expressed as relative luciferase units.

### Immunoprecipitation

H1819 cells were harvested and lysed in ice-cold RIPA buffer (1×10^7^cells/ml) after treated with or without rhEPO (10IU/ml). After centrifuge at 4°C for 5 min, cell lysate were pre-cleaned with 50% Protein A magnetic bead slurry (Cell Signaling Technology, CST, Beverly, MA). The phospho-JAK2 antibody (CST) was added to 200 μl cell lysate at 100 μg/ml, and incubated with rotation overnight at 4 °C. Protein A magnetic beads (10–30 μl of 50% bead slurry) were used to pull down the target proteins. The levels of EPOR (ab56310, Abcam) and JAK2 (ab39636, Abcam) in the pellet were analyzed by western immunoblotting.

### Chromatin immunoprecipitation (ChIP) assay

ChIP assay was performed on H1819 cells by using a mouse anti-STAT5a antibody (ab32043, Abcam). Pre-immune mouse serum was used as a negative control. Two primer sets were designed to flank two putative STAT5a binding sites in the promoter region of CCND1. Cells were sonicated and fixed with 1% paraformaldehyde for pull-down of chromatin associated with CCND1 using STAT5a antibody or control IgG. The amount of the specific DNA fragment was then quantified by qRT-PCR.

### Statistical analysis

Mann-Whitney U test was used to examine associations between EPO and EPOR expression in human samples and clinical variables. Kaplan-Meier survival curves and log rank tests were used to examine the association between tumor expression of EPO or EPOR and patient overall survival. Statistical comparisons between patients’ serum EPO levels before and after surgeries were analyzed by paired Student’s t test. For *in vitro* studies, all experiments were performed in triplicate, and the continuous variables were compared using the unpaired Student’s t test if normally distributed. For animal experiments, six mice were assigned to each treatment group. This sample size gave 80% power to detect a 50% reduction in tumor weight with 95% confidence. The growths of xenograft tumors were compared by two-way ANOVA. For analyzing the correlation, we used two sided Pearson’s test. All data are presented as mean ± SD, and *P* values ≤ 0.05 were considered significant for all analyses.

The full experimental procedures are described in the Supplemental Materials.

## SUPPLEMENTARY MATERIALS FIGURES AND TABLE



## Abbreviations

EPO: erythropoietin
EPOR: erythropoietin receptor
NSCLC: non-small cell lung cancer
rhEPO: recombinant human erythropoietin
ESAs: erythropoiesis stimulating agents
TMA: tissue microarrays
ELISA: enzyme-linked immunosorbent assay
EPO-NA: EPO neutralizing antibody
shRNA: short hairpin RNA
siRNA: short interfering RNA
JAK2: Janus kinase 2
STAT5: signal transducer and activator of transcription 5
AKT: protein kinase B
MAPK: mitogen-activated protein kinases
HIF-1α: hypoxia-inducible factor 1-alpha
