# Supplementary material for: Local blockage of self-sustainable erythropoietin signaling suppresses tumor progression in non-small cell lung cancer
Source: Oncotarget. 2017 Jul 18;8(47):82352–65. doi: 10.18632/oncotarget.19354 (PMC5669895; doi:10.18632/oncotarget.19354)
Supplement: Supplementary file 1 [file oncotarget-08-82352-s001.pdf]

Local blockage of self-sustainable erythropoietin signaling suppresses tumor progression in non-small cell lung cancer

SUPPLEMENTARY MATERIALS

Immunohistochemistry (IHC)

Immunochemical staining was performed on 5 µm-thick sections of FFPE specimens and tissue microarrays using the following primary antibodies: EPO (AB-286-NA, R&D, Minneapolis, MN), EPOR (ab56310, Abcam, Cambridge, UK), HIF1α (ab16066, Abcam), JAK2 (ab39636, Abcam), STAT5 (#9363, Cell Signaling Technology, CST, Beverly, MA) or Cyclin D1 (MAB4314, R&D). The staining procedure was based on the manufacturer's instruction of an IHC commercial kit (TANGEN, Shanghai, China). The sections were counterstained with Harris' hematoxylin. The staining was evaluated blindly by two observers with a scoring system as follows: 0, negative expression; 1+, moderately positive expression; 2+, strongly positive expression. The specimens of mice xenograft tumor were stained for Ki67 (ab15580, Abcam). The Ki67 labeling index was evaluated and determined also blindly by two observers.

Cell proliferation, cell cycle and apoptosis assay

Cells were treated by rhEPO (R&D) or by NSCLC patients' serum in the presence or absence of an EPO neutralizing antibody (EPO-NA, 5µg/ml, AB-286-NA, R&D). The working concentration of EPO-NA (5µg/ml) was determined according to the ELISA analysis of the patients' and volunteers' sera. An absolutely excessive amount (5µg/ml) was applied to assure the complete blockage of EPO in patients' sera. The sera were pre-incubated with EPO-NA for 30 min at 37°C before applying to cells. Cell proliferation was determined by Thiazolyl Blue Tetrazolium Bromide (MTT) method (Sigma-Aldrich, St. Louis, MO). Cell-cycle analysis was performed by propidium iodide (PI) staining or by EdU incorporation using Click-iT EdU Alexa Fluor 647 Flow Assay Kit (Invitrogen, Carlsbad, CA) following the manufacturer's instructions. For apoptosis assay, cells were treated with etoposide (50 µM) or etoposide in combination with rhEPO (10 IU/ml). The cells were then stained with FITC Annexin V and PI (Invitrogen). Cell cycle and apoptosis were analyzed by a CyAnTM ADP flow cytometer (Dako, Glostrup, Denmark). Data analysis was performed using FlowJo (Tree Star Inc., Ashland, OR).

Real-time quantitative PCR

Total RNA was isolated using TRIzol reagent (Life Technologies, Gaithersburg, MD) and then reverse-transcribed using iScript cDNA Synthesis Kit (Bio-Rad, Hercules, CA). The real-time PCR was performed using iTaq Universal SYBR Green Supermix (Bio-Rad) for the following target genes with primers listed below. The β actin was included as reference gene for each reaction.

	Sense	Anti-sense
EPO	GATAAAGCCGTCAGTGGCCTTC	GGGAGATGGCTTCCTTCTGGG
EPOR	CCTGACGCTCTCCCTCATCC	GCCTTCAAACTCGCTCTCTGG
Cyclin D1	TGAGGCGGTAGTAGGACAGG	GACCTTCGTTGCCCTCTGT
Cyclin D2	ACGGTACTGCTGCAGGCTAT	AGCTGCTGGCTAAGATCACC
Cyclin D3	TTGAGCTTCCCTAGGACCAG	TGACCATCGAAAAACTGTGC
Cyclin A	CACTCACTGGCTTTTCATCTTC	CAGAAAACCATTGGTCCCTC
HIF1α	TGGCTGCATCTCGAGACTTT	GAAGACATCGCGGGGAC
β Actin	AGAGGGAAATCGTGCGTGAC	CAATAGTGATGACCTGGCCGT

Western blot

Cells or flash-frozen tumor tissues were washed with PBS and harvested in RIPA lysis buffer (Life Technologies). Protein concentration was determined with Bradford protein assay (Bio-Rad). Equal amounts of protein were separated on SDS-PAGE and transferred to PVDF membranes (Bio-Rad). Membranes were blocked with 5% skim milk and

incubated at 4°C overnight with a primary antibody. The membranes were then probed with secondary antibodies, and immunoreactions were detected with ECL substrate (Bio-Rad). GAPDH or β-actin was used as loading control. The information of antibodies are listed below.

Pharmacological inhibitors

The source information of JAK2, STAT5, PI3k/Akt, MAPK and HIF-1α inhibitors are listed below.

Antibody	Cat.No.	Manufacturer
EPO	ab126876	Abcam
EPOR	ab56310	Abcam
phospho-EPOR	MAB6926	R&D
JAK2	ab39636	Abcam
phospho-JAK2	#3771	CST
STAT5	ab68465	Abcam
phospho-STAT5	#9314	CST
STAT2	#4594	CST
phospho-STAT2	#4441	CST
STAT3	#9139	CST
phospho-STAT3	#9145	CST
STAT5a	ab32043	Abcam
phosphor-STAT5b	ab52211	Abcam
AKT	#9272	CST
phospho-AKT	#4060	CST
p38	#8690	CST
phospho-p38	#4511	CST
Cleaved caspase 3	#9662	CST
Cyclin D1	MAB4314	Abcam
Cyclin D2	#3741	CST
Cyclin D3	684902	BioLegend
Cyclin A	644001	BioLegend
HIF1α	ab51608	Abcam
GAPDH	ab8245	Abcam
β-actin	ab8226	Abcam

Inhibitors	Cat.No.	Manufacturer
AG490 (JAK2)	658411	Merk Millipore
CAS285986 (STAT5)	573108	Merk Millipore
LY294002 (PI3k/Akt)	440204	Merk Millipore
PD98059 (p38 MAPK)	513000	Merk Millipore
YC1 (HIF1α)	170632-47-0	Sigma-Aldrich

Gene knockdown with small interfering RNAs (siRNAs) or short hairpin RNA (shRNA)

The siRNAs against EPOR, STAT5, JAK2 and their corresponding controls were designed and manufactured by GenePharma Co, Ltd. (Pudong, Shanghai, China). The sequences for these siRNAs and controls are listed below.

		Sense	Anti-sense
EPOR	siEPOR	CCGUGUCAUCCACAUCAAUTT	AUUGAUGUGGAUGACACGGTT
	siCON	GGUTTTAUGCCGAUTTAACGAT	GTUAUAATTCGCGCCUTTAUTT
STAT5	siSTAT5	CCGGCACAUUCUGUACAAUTT	AUUGUACAGAAUGUGCCGGTT
	siCON	CAUTUGGCCUTUTAACGUATT	ACCUCGAUTACUGGCGUAATT
JAK2	siJAK2	CCACCUGAAUGCAUUGAAATT	UUUCAAUGCAUUCAGGUGGTT
	siCON	CUAGCUAGGTTUCCAUTCCTT	AUCGCCUTTCGGUAUAUTCTT

Lipofectamine 2000 Reagent (Life Technologies) was used to transfect nucleic acids into NSCLC cells following the manufacturer's instructions. The expression of EPOR, JAK2 and STAT5 was determined by qRT-PCR and western blot within 72 hours. The shRNA expression constructs against EPOR and control plasmid were purchased from GeneCopoeia (Rockville, MD). NSCLC cells were transfected with the constructs using FuGENE HD Transfection Reagent (Roche, Basel, Switzerland). The stable clones were distinguished by continuous selection with Hygromycin B (3-4 weeks) (Roche). The expression of EPOR was determined by qRT-PCR and western blot.

Immumofluorescent staining

H1819 cells were grown in 4-chamber slides (Sigma Aldrich) to 80% confluence and treated with or without rhEPO (10 IU/ml). The cells were then fixed in 4% paraformaldehyde (PFA), blocked with 20% goat serum and incubated with STAT5a antibody (ab32043, Abcam) or STAT5b antibody (ab178941, Abcam) in 4°C overnight. After incubation with a FITC-tagged secondary antibody followed by counterstaining with DAPI, images were collected using a confocal fluorescence microscope (Fv1000, OLYMPUS, Tokyo, Japan).

Cell migration assay

Cell migration under rhEPO treatment was assessed using 24-well trans-well units with 8 μm pore size polycarbonate inserts (Corning Life Sciences, NY). Briefly, the wells were coated with Basement Membrane Extract (Life Technologies) according to the manufacturer's instructions. NSCLC cells were plated into coated wells with serum-free medium at $1x10^5$ cells/well. The rhEPO (10IU/ml) were added to the lower chamber of the trans-well units and the wells were incubated in an incubator for 48 hours at 37 °C. The cells on the bottom of the upper chamber were counted after fixation and crystal violet staining. Transforming growth factor β (TGFβ) (0.5μg/ml) (Life Technologies) treatment was included as a positive control.

Hypoxic treatments of cultured cells

Hypoxia treatment was performed in a hypoxic cell culture system (HERACELL 150i, Thermo Scitntific). Cells were exposed to 1% O2 for 24 hours before analyzing EPO mRNA expression and protein secretion. For cell proliferations, the hypoxia treatment duration was 48 hours. For RNA interference experiments, the EPOR siRNA was transfected into cells at 24 hours before the application of hypoxia treatment. The details about qRT-PCR, ELISA, Edu incorporation and siRNA interference were provided in the corresponding method sections.

Supplementary Table 1: Demographic and clinical characteristics of NSCLC patients based on EPO and EPOR exp ressions

	No. of patients (n)	EPO (n)				EPOR (n)			
		0	1+	2+	*P*	0	1+	2+	*P*
**All patients (n=60)**		22	17	21	/	4	31	25	/
**Age**									
61<	26	10	9	7	0.437	3	10	13	0.518
61≥	34	12	8	14		1	21	12	
**Gender**									
male	36	13	11	12	0.904	2	17	17	0.285
female	24	9	6	9		2	14	8	
**Smoking status**									
non-smoker	21	11	6	4	0.035	3	10	8	0.393
current or ex-smoker	39	11	11	17		1	21	17	
**Histology**									
adenocarcinoma	30	11	8	11	0.881	2	17	11	0.485
squamous cell carcinoma	30	11	9	10		2	14	14	
**Stage**									
II	27	17	5	5	0.001	7	8	12	0.097
III+IV	33	5	12	16		15	9	9	

0, negative expression; 1+, weakly and moderately positive expression; 2+, strongly positive expression. Mann-Whitney U test was used to compare the paitnets' distribution in groups. All p values are two-sided. Cut off value of age is median value.

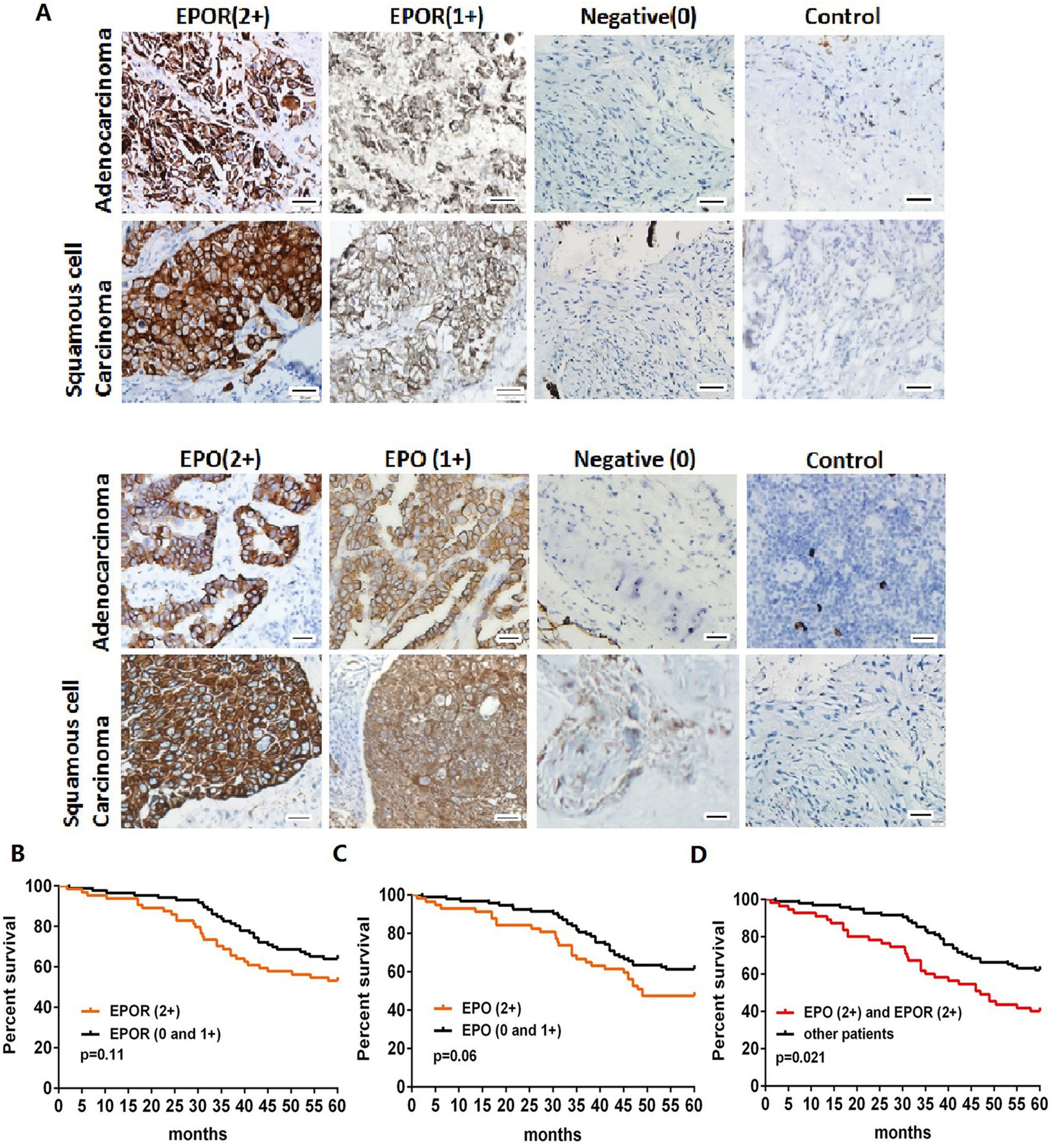

Supplementary Figure 1: The expression of erythropoietin (EPO) and EPO receptor (EPOR) in non-small cell lung cancer (NSCLC) are associated with tumor progression and overall survival. **(A)** Representative immunohistochemistry images of EPOR (upper panel) and EPO (lower panel) on squamous cell carcinomas and adenocarcinomas. **(B and C)** Kaplan-Meier curves of overall survival for 150 NSCLC patients stratified by EPO or EPOR expression levels in tumors. The single strong positive group (EPO 2+ or EPOR 2+) had a poor five-year overall survival (p=0.06 for EPOR and p=0.11for EPO). **(D)** The double strong positive group (EPO 2+/EPOR2+) had a significant poorer 5-year overall survival compared with other patients (p=0.021). Bar=20 μm.

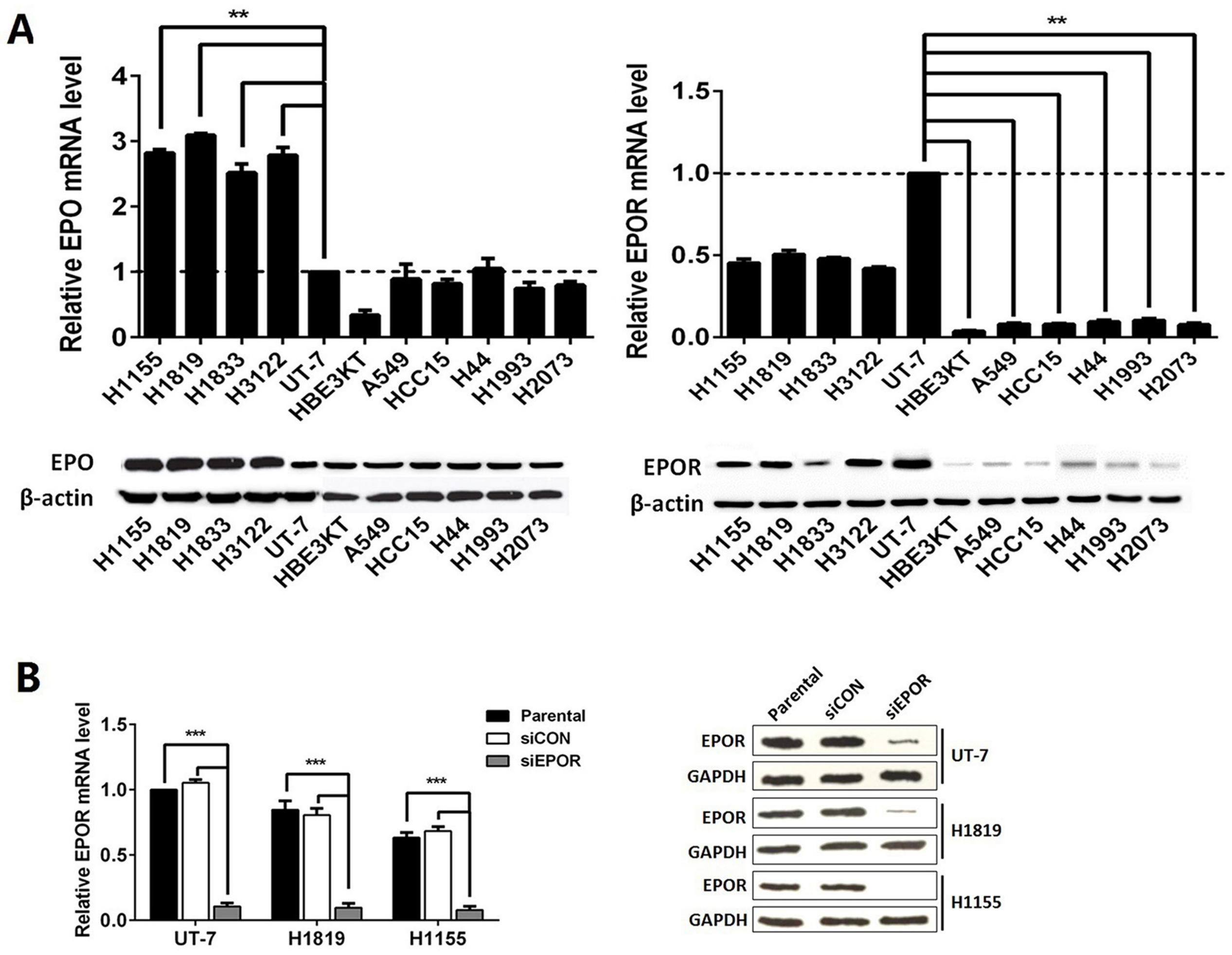

Supplementary Figure 2: The expression of erythropoietin (EPO) and EPO receptor (EPOR) in non-small cell lung cancer cell lines. (A) Comparative analysis of EPO and EPOR mRNA (upper panels) and protein (lower panels) expression in NSCLC cells. The EPO-dependent leukemia cells UT-7 and normal bronchial epithelial cells HBEC-3KT were included as controls. **(B)** Treatment by siRNAs against EPOR (siEPOR) significantly reduced EPOR mRNA and protein expression in UT-7, H1155 and H1819 cells. Mean±SD, ***, p≤0.001; **, p≤0.01.

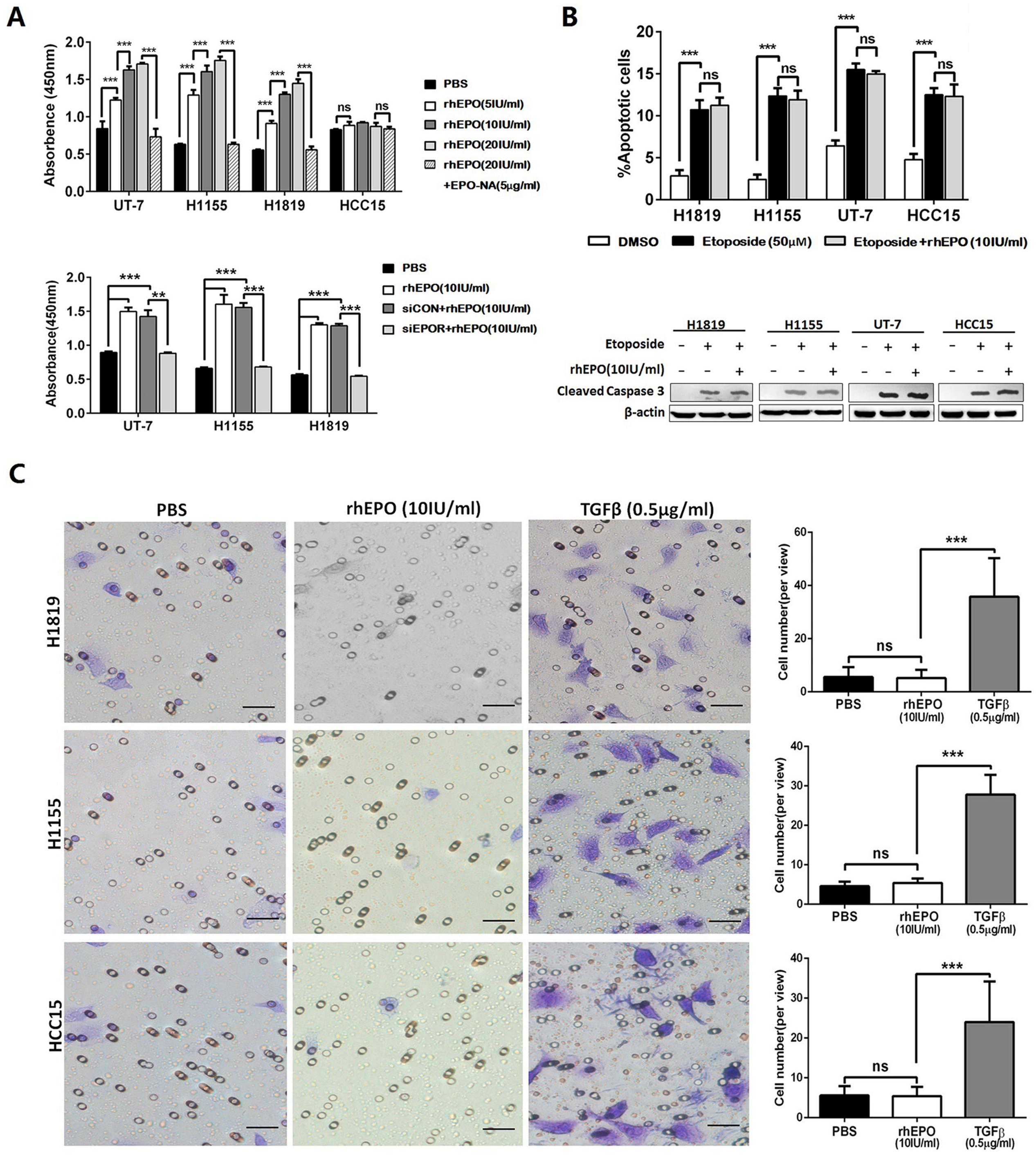

Supplementary Figure 3: Recombinant human erythropoietin (rhEPO) promoted cell proliferation I NSCLC cells. **(A)** Treatment by rhEPO produced the growth-promoting effects like in patients' sera on UT-7, H1155 and H1819 cells, which was totally blocked by EPO neutralizing antibody (EPO-NA, upper panel) or by siRNA against EPOR (siEPOR, lower panel). The rhEPO had no significant growth-promoting effects in HCC15 cells. **(B)** Flow cytometry (Annexin V and PI staining) and Western blots for cleaved caspase 3 showed rhEPO did not relieve the etoposide-induced apoptosis of NSCLC cells. **(C)** Trans-well assay showed that rhEPO did not promote the cell migrations of NSCLC cells. Transforming growth factor β (TGF β) were used as a positive control. Bar=20μm; Mean±SD, ***, p≤0.001; ns, not significant.

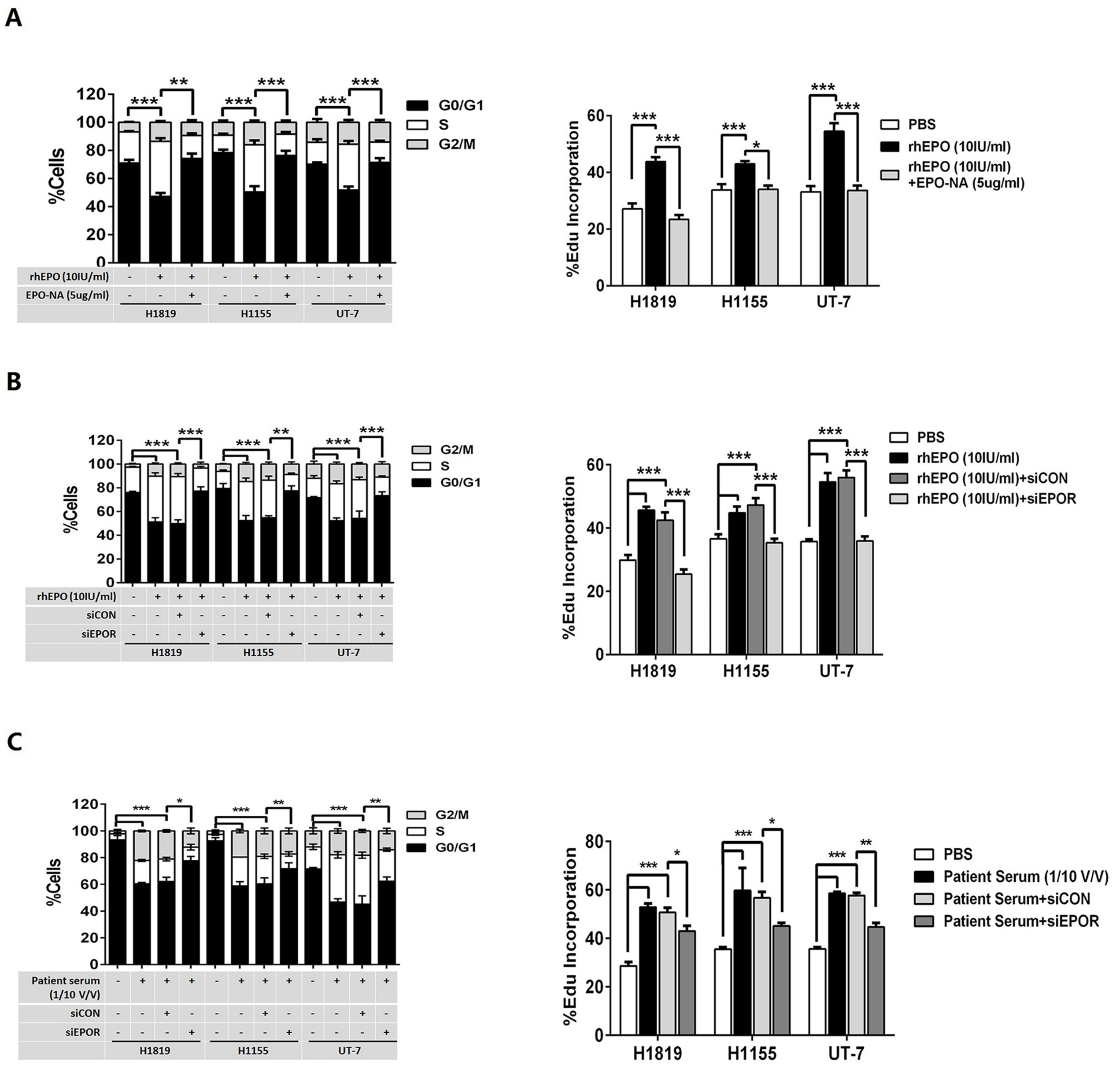

Supplementary Figure 4: EPO/EPOR signaling promoted cell cycle in NSCLC cells. (A) rhEPO increased percentage of S-phase cells as well as EdU incorporation in H1819, H1155 and UT-7. The EPO neutralizing antibody (EPO-NA) diminished the cell cycle-promoting effect of rhEPO. **(B)** Treatment by siRNA against EPOR (siEPOR) diminished the cell cycle promotion effects of rhEPO in H1819, H1155 and UT-7. **(C)** Treatment by the sera from NSCLC patients promoted cell cycle of NSCLC cells, and siEPOR partially abolished this promotion effects. Mean±SD; ***, p≤0.001; **, p≤0.01; *, p≤0.05.

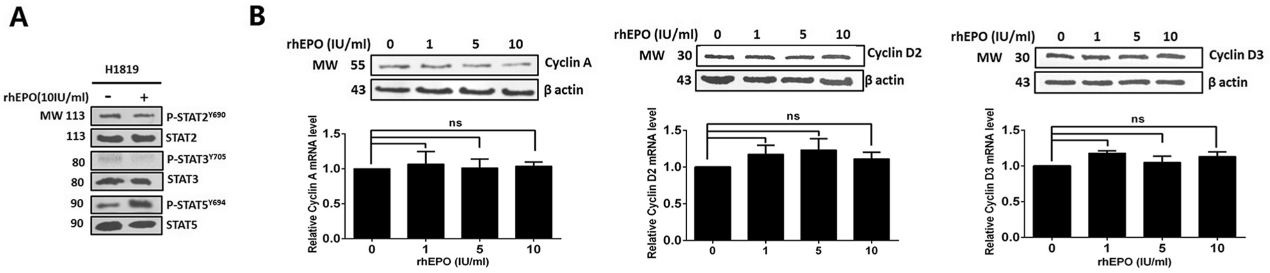

C

-1500 GGGAGGGGGCGCGGGAGGAATTCACCCTGAAAGGTGGGGGTGGGGGGGGTCGCATCTTGCTGTGAGCACCCTGGCGAAGGGGAGAGGGCTTTTTCTATCA
-1400 GTTTTCTTTGAGCTTTTACTGTTAAGAGGGTACGGTGGTTTGATGACACTGAACTATATTCAAAAGGAAGTAAATGAACAGTTTTCTTAATTTGGGGCAG
-1300 GTACTGTAAAAATAAAAACAAAAGTTAAGACAGTAAAATGTCCTTTTATTTTTTAATGCACCAAAGAGACAGAACCTGTAATTTTAAAAACTGTGTATTT
-1200 TAATTTACATCTGCTTAAGTTTGCGATAATATTGGGGACCCTCTCATGTAACCACGAACACCTATCGATTTTGCTAAAAATCAGATCAGTACACTCGTTT
-1100 GTTTAATTGATAATTGTTCTGAATTATGCCGGCTCCTGCCAGCCCCCTCACGCTCACGAATTCAGTCCCAGGGCAAATTCTAAAGGTGAAGGGACGTCTA
-1000 CACCCCCAACAAAACCAATTAGGAACCTTCGGTGGTCTTGTCCCAGGCAGAGGGGACTAATATTTCCAGCAATTTAATTTCTTTTTTAATTAAAAAAAAT
-900 GAGTCAGAATGGAGATCACTGTTTCTCAGCTTTCCATTCAGAGGTGTGTTTCTCCCGGTTAAATTGCCGGCACGGGAAGGGAGGGGGTGCAGTTGGGGAC
-800 CCCCGCAAGGACCGACTGGTCAAGGTAGGAAGGCAGCCCGAAGAGTCTCCAGGCTAGAAGGACAAGATGAAGGAAATGCTGGCCACCATCTTGGGCTGCT
-700 GCTGGAATTTTCGGGCATTTATTTTATTTTATTTTTTGAGCGAGCGCATGCTAAGCTGAAATCCCTTTAACTTTTAGGGTTACCCCCTTGGGCATTTGCA
-600 ACGACGCCCCTGTGCGCCGGAATGAAACTTGCACAGGGGTTGTGTGCCCGGTCCTCCCCGTCCTTGCATGCTAAATTAGTTCTTGCAATTTACACGTGTT
-500 AATGAAAATGAAAGAAGATGCAGTCGCTGAGATTCTTTGGCCGTCTGTCCGCCCGTGGGTGCCCTCGTGGCGTTCTTGGAAATGCGCCCATTCTGCCGGC
-400 TTGGATATGGGGTGTCGCCGCGCCCCAGTCACCCCTTCTCGTGGTCTCCCCAGGCTGCGTGTGGCCTGCCGGCCTTCCTAGTTGTCCCCTACTGCAGAGC
-300 CACCTCCACCTCACCCCCTAAATCCCGGGGGACCCACTCGAGGCGGACGGGGCCCCCTGCACCCCTCTTCCCTGGCGGGGAGAAAGGCTGCAGCGGGGCG
-200 ATTTGCATTTCTATGAAAACCGGACTACAGGGGCAACTCCGCCGCAGGGCAGGCGCGGCGCCTCAGGGATGGCTTTTGGGCTCTGCCCCTCGCTGCTCCC
-100 GGCGTTTGGCGCCCGCGCCCCCTCCCCCTGCGCCCGCCCCCGCCCCCCTCCCGCTCCCATTCTCTGCCGGGCTTTGATCTTTGCTTAACAACAGTAACGT
+1
CACACGGACTACAGGGGAGTTTTGTTGAAGTTGCAAAGTCCTGGAGCCTCCAGAGGGCTGTCGGCGCAGTAGCAGCGAGCAGCAGAGTCCGCACGCTCCGGCGAGGGGCAGAAGAGCGCGAGGGAGCGC
GGGGCAGCAGAAGCGAGAGCCGAGCGCGGACCCAGCCAGGACCCACAGCCCTCCCCAGCTGCCCAGGAAGAGCCCCAGCCATGCACCAGCTCCTGTGCTGCGAAGTGGAAACCATCCG

Supplementary Figure 5: Recombinant human erythropoietin (rhEPO) treatment activated STAT5 and CCND1-specific pathway in NSCLC cells. **(A)** The rhEPO treatment only increased the phosphorylation of STAT5 in H1819. **(B)** The rhEPO treatment did not alter expressions of cyclin D2, cyclin D3 and cyclin A. **(C)** Two putative STAT5a-binding elements within the promoter fragment (1.5kb) of cyclin D1 (CCND1) identified using online algorithms (-1401 and -194). Mean±SD; ns, not significant.

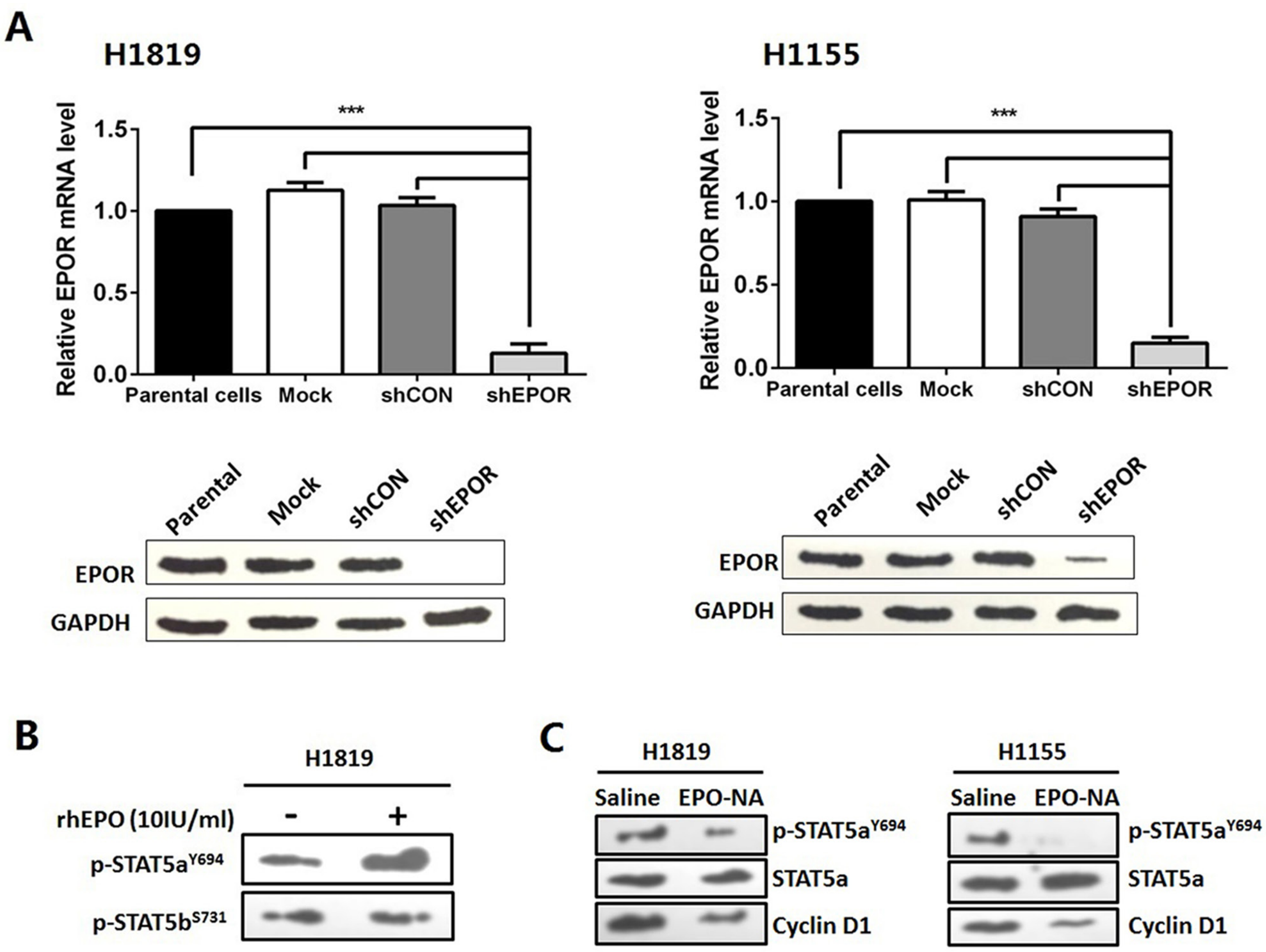

Supplementary Figure 6: EPO/EPOR signaling was through a Stat5a-dependent pathway in NSCLC. (A) The rhEPO treatment induced the phosphorylation of STAT5a but not that of STAT5b. **(B)** The mRNA and protein expression of EPOR were significantly reduced in H1819 and H1155 cells after stable transfection with short hairpin RNA against EPOR. **(C)** The phosphorylation of STAT5a and the expression of cyclin D1 in EPO-NA (EPO neutralizing antibody) treated H1819 and H1155 xenograft tumors significantly reduced compared with those treated by normal saline. Mean±SD; ***, $p \leq 0.001$.
